# Dynamic self-organization of migrating cells under constraints by spatial confinement and epithelial integrity

**DOI:** 10.1140/epje/s10189-022-00161-x

**Published:** 2022-02-25

**Authors:** Tetsuya Hiraiwa

**Affiliations:** 1 Mechanobiology Institute, National University of Singapore, Singapore, Singapore 117411; 2 Universal Biology Institute, The University of Tokyo, Hongo, Tokyo, 113-0033 Japan

## Abstract

**Abstract:**

Understanding how migrating cells can establish both dynamic structures and coherent dynamics may provide mechanistic insights to study how living systems acquire complex structures and functions. Recent studies revealed that intercellular contact communication plays a crucial role for establishing cellular dynamic self-organization (DSO) and provided a theoretical model of DSO for migrating solitary cells in a free space. However, to apply those understanding to situations in living organisms, we need to know the role of cell–cell communication for tissue dynamics under spatial confinements and epithelial integrity. Here, we expand the previous numerical studies on DSO to migrating cells subjected spatial confinement and/or epithelial integrity. An epithelial monolayer is simulated by combining the model of cellular DSO and the cellular vertex model in two dimensions for apical integrity. Under confinement to a small space, theoretical models of both solitary and epithelial cells exhibit characteristic coherent dynamics, including apparent swirling. We also find that such coherent dynamics can allow the cells to overcome the strong constraint due to spatial confinement and epithelial integrity. Furthermore, we demonstrate how epithelial cell clusters behave without spatial confinement and find various cluster dynamics, including spinning, migration and elongation.

**Graphical abstract:**

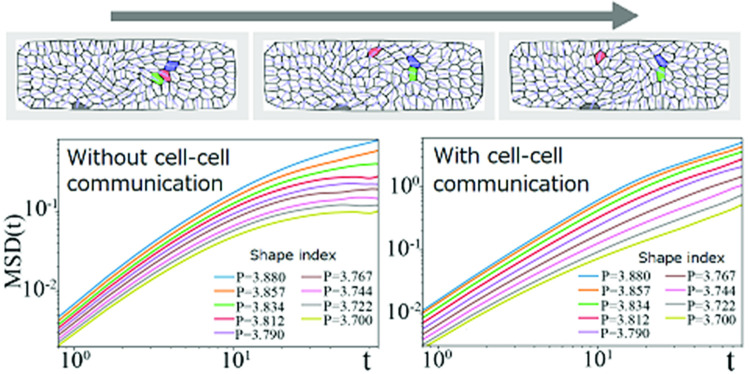

## Introduction

The emergent mechanisms by which living systems acquire complex structures and functions have not been completely understood yet. Recently, the importance of self-organization processes in biological systems has been recognized not only in the physics community, but also in biology community [1–3]. Living systems need to demonstrate emergence of dynamic properties such as coherent dynamics and formation of dynamic patterns, as well as static properties. For example, cells often move cooperatively and spontaneously organize their coherent dynamics [4–12]. Accumulated experimental knowledge suggested that intercellular contact communication plays crucial roles for such dynamic self-organization of migrating cells [11, 12], and the theoretical model which can recapture those dynamic phenomena in solitary cells has been provided [13, 14].

**Fig. 1:**
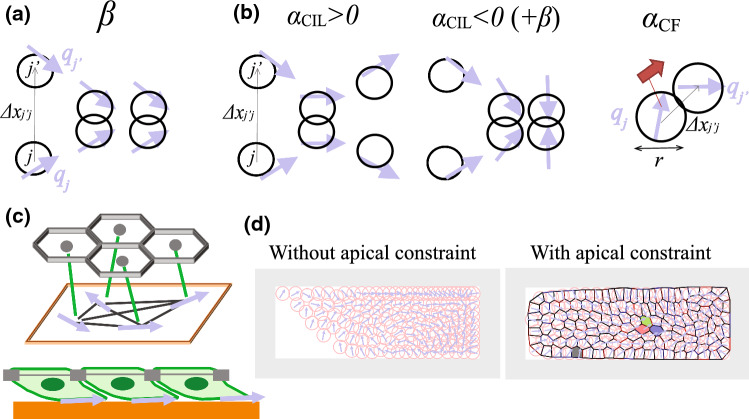
Mathematical model investigated in this article. **a**, **b** Cell–cell interactions assumed in this article, including **a** volume exclusion and **b** cell–cell communication through basal side. In particular, we take into account (**b**; left) contact inhibition of locomotion, (**b**; center) contact attraction of locomotion and (**b**; right) contact follow of locomotion. **c** Schematic illustration of the case with epithelial integrity. **d** Sample snapshots of numerical simulations based on this model. Circles indicate the cell periphery (i.e., half interaction range) at the basal side, and arrows represent the intrinsic polarity ${\varvec{q}}$ of each cell

One of the next challenges will be to investigate how dynamic self-organization occurs in living organisms. In multicellular living organisms, establishment of coherent dynamics can occur not only by freely-migrating solitary cells, but also by cells under various constraints. An important class of such cells is epithelial cell monolayers, where cells are tightly packed in a sheet-like tissue without gaps and adheres each other mechanically. A classic example of an epithelial cell line which exhibits this organization is Madin-Darby Canine Kidney (MDCK) cells [15, 16]. It is indeed known that such epithelial cells exhibit coherent dynamics [15, 16], which can be relevant to *in situ* biological processes like gastrulation [17]. Uncovering the mechanisms by which coherent migratory dynamics occur in epithelial cells may provide us new ways to understand mechanistic aspects in such biological processes. In addition to this, as living organisms have a finite size, it is important to consider spatial confinement as a constraint for establishing coherent dynamics. However, still there is a lack of knowledge regarding how cell–cell contact communication can contribute to dynamics of migrating cells under these constraints.

Here, we aim to expand on our earlier work demonstrating theoretical understanding of dynamic self-organization of migrating cells through cell–cell contact communication [14] to cases where migrating cells are subject to constraints of spatial confinement and/or epithelial integrity, i.e., mechanical constraint due to the integrity of an epithelial tissue. After investigating coherent dynamics in cells under spatial confinement but without epithelial integrity, we extended the study to investigate coherent dynamics under epithelial integrity. We assume that the cells interact with each other through mechanical mutual exclusion and two ubiquitous types of cell–cell contact communication, namely contact following and contact inhibition of locomotion [13, 14]. For epithelial cell’s case, numerical simulations are performed based on the mathematical model of cell migration in an epithelial tissue using the following assumptions: (i) Each cell consists of apical and basal sides. (ii) Epithelial integrity at the apical side is modeled based on the cellular vertex model in two dimensions [18], and for brevity the constraints due to this vertex model at the apical side are termed apical constraint. (iii) The basal side of each cell is modeled by a single disk which spontaneously move on the two-dimensional substrate according to its intrinsic polarity [19]. (iv) The apical and basal sides of each cell are connected to each other through a linear elastic spring. (v) Cell–cell contact communication as mentioned above takes place through the basal sides of cells.

Before closing this section, we make comments regarding with a brief review theoretical modeling. Epithelial tissues play an essential biological roles in shaping the multicellular body. Based on this important role, a model for migrating epithelial cells which implements the polygonal shape of cells by Voronoi tessellation (using the points which spontaneously move around), later termed the self-propelled Voronoi model, has been proposed in Ref. [20], and this model was further investigated in details from the perspective of statistical physics [21]. By assuming the tendency that cell polarity direction aligns to the actual direction of migration, flocking behavior was also investigated using this model [22]. Variations on this self-propelled Voronoi model have also been proposed. One example is based on Delaunay triangulation instead of Voronoi tessellation [23]. Furthermore, models combining spontaneous cell motility with purely the vertex description, which does not assume the cell shapes described by Voronoi volumes, have been introduced [24, 25]. These concepts, i.e., a combination of spontaneous motility and polygonal shape description of cells, may also be a powerful tool to study cellular dynamic self-organization in an epithelial tissue, where the cells interact through the apical connections. However, for this aim, the motility of the basal side should be appropriately described; it is not the subordinate of the apical configuration, but can show independent migratory activity [26]. Therefore, the model used in this article takes into account such mutual independence of apical and basal dynamics by assuming both of them to be independent variables.

This article is organized as follows: The theoretical model which we investigate in this article is described in Sect. 2, with numerical results in Sect. 3. In particular, we investigate the cases of freely migrating cells under confinement, migrating epithelial cells (i.e., cells with apical constraints) under confinement, and migrating epithelial cells in a free space in Subsects. 3.1, 3.2, and 3.3, respectively. Section 4 is devoted to a brief summary and discussion. “Appendix A” provides the numerical results for freely migrating cells in a periodic system boundary, similar to Ref. [14] but with a smaller system size and a different set of parameters. “Appendix B” provides the numerical results of the case with apical junctional tension fluctuation. “Appendix C” provides the comprehensive discussion on how we determine the state boundaries in this article.

## Model

As described in Sect. 1 and Fig. 1, we mathematically model the population of migrating cells by mechanical mutual exclusions (Fig. 1a) and intercellular communication (Fig. 1b) on a two-dimensional flat substrate. Later, in part, we consider an epithelial monolayer in which each cell is spontaneously migrating (Fig. 1c). The basal side of each cell is modeled by a so-called self-propelled particle, meaning that the particle is spontaneously moving on the substrate according to its intrinsic polarity [14, 19]. This mimics the spontaneous migration ability of each cell on the basal substrate. The apical side of the cell is modeled based on the vertex dynamics framework in two dimensions, which can simulate in-plane motions of cells in an epithelial monolayer tissue [18]. In this framework, since the individual cells in an epithelial monolayer often have straight cell–cell junctions at the apical face, the shape of each cell is described by a polygonal shape. The apical and basal sides of a cell are connected with each other through a linear elastic spring (Fig. 1c). The system is assumed to have a boundary over which the cells cannot travel (Fig. 1d), except when considering migrating cells in a free space (Sect. 3.3). We adopt the rectangular boundary with the aspect ratio of 20/7 for this, except for Sect. 3.3.

This article aims at investigating the impact of contact intercellular communication for DSO of migrating cells under constraints. In particular, we focus on the following manners of intercellular communication, called contact inhibition/attraction of locomotion (CIL/CAL) and contact following (CF), as with our previous work [14]. *CIL:* When two cells come in contact, they modulate their polarities to avoid overlap and migrate away as if they are scattered (Fig. 1b left) [5, 6, 27–29]. This is called CIL. It is known that, for example, CIL takes place in *Xenopus* neural crest cells [5] and is involved in coordinated migration and directional migration in response to external cues [6, 27]. *CAL:* When two cells come in contact, they modulate their polarity such that they are mutually attracted (Fig. 1b center) [8]. This is called CAL and may result in their behaviors in population. *CF:* When a cell contacts another cell, the cell at the back chases the cell at the front but not vice versa (Fig. 1b right). This is called CF and has been observed in several cells [11, 12, 30]. CF is involved in their DSO, such as formations of rotating mount [11] and traveling density band [12].

We assume that, except for the self-propulsion mechanism at the basal side, the system obeys variational dynamics with the potential function *E* composed of three parts

1 $$\begin{aligned} \begin{aligned} E( \{\varvec{r}_i \}, \{\varvec{X}_{\alpha } \})&= E_\mathrm{vertex}( \{\varvec{r}_i \}) + E_\mathrm{VE}( \{\varvec{X}_{\alpha } \}) \\&\quad + E_\mathrm{A-B}( \{\varvec{r}_i \}, \{\varvec{X}_{\alpha } \}) \ . \end{aligned} \ \end{aligned}$$

Each contribution in the potential, $E_\mathrm{vertex}$ for the apical side, $E_\mathrm{VE}$ for the basal side and $E_\mathrm{A-B}$ for the apico-basal connection, is explained in detail in the following subsections. Here, $\varvec{r}_i$ ($i=1,2,\ldots ,M$ with the total number of vertices *M*) and $\varvec{X}_{\alpha }$ ($\alpha =1,2,\ldots ,N$ with the number of cells *N*) denote the variables specifying the configurations at the apical and basal sides of cells, respectively, the details of which are also explained in the following subsections.

This manner of implementation of epithelial integrity, for which we introduce two independent layers of variables, reflects the experimental observations for dynamics of apical and baso-lateral sides of a cell, which can be independent with each other, as described in Introduction. This implementation may inherently be able to cause characteristic dynamics replying on the relative motion of apical and basal sides of each cell. However, we have not observed such characteristic dynamics requiring two layers of variables so far. Moreover, in this article, we implement epithelial integrity by the vertex model, following the convention for the simulations of epithelial tissue dynamics. However, the integrity can be also implemented using the disk-disk adhesion [12]. It might be another interesting question how this difference in implementation of cell–cell adhesion can affect the results.

### Model for basal motility: Self-propelled particle model

For this part, we consider *N* particles in the two-dimensional space surrounded by the system boundary. The particle with the indices ${\alpha }$ (${\alpha }=1,2, \ldots , N$) represents the basal body of the ${\alpha }$-th cell, and the location of it is described by ${\varvec{X}}_{\alpha } =(X_{\alpha }, Y_{\alpha })$. The velocity ${\varvec{v}}_{\alpha } \equiv d {\varvec{X}}_{\alpha }/dt$ and the intrinsic polarity ${\varvec{q}}_{\alpha }$ of the ${\alpha }$-th particle obey

2 $$\begin{aligned} \frac{\mathrm{d} {\varvec{X}}_{\alpha }}{\mathrm{d}t} = \varGamma \left( e \hat{\varvec{q}}_{\alpha } - \frac{\partial E}{\partial {\varvec{X}}_{\alpha }} \right) \ , \end{aligned}$$

and with $$\hat{\varvec{q}}_{\alpha }={\varvec{q}}_{\alpha }/|{\varvec{q}}_{\alpha }|$$

3 $$\begin{aligned} \frac{\mathrm{d} {\varvec{q}}_{\alpha }}{\mathrm{d}t} = I_q (Q^2-|{\varvec{q}}_{\alpha }|^2) {\varvec{q}}_{\alpha } + {\varvec{J}^{q}}_{\alpha } + {\varvec{\xi }^{q}}_{\alpha } \ , \end{aligned}$$

respectively [12–14, 31, 32].

Equation (2) is the equation of motion, where $\varGamma$ is the mobility coefficient and *e* is the coefficient representing effective driving force for migration, and the last term is the mechanical interaction between the particles with the potential *E* in Eq. (1). In the potential *E*, the parts relevant for the basal side dynamics are those for the volume exclusion $E_\mathrm{VE}$ (Fig. 1a) and for the apico-basal connection $E_\mathrm{A-B}$ (Fig. 1c). (For $E_\mathrm{A-B}$, see Subsect. 2.3.) For simplicity, we idealize each cell as a soft-core repulsive disk with a fixed diameter *r*, thus assume

4 $$\begin{aligned} \begin{aligned} E_\mathrm{VE}&= \beta ' r \sum _{<{\alpha }, {\alpha }'>} \left[ -\log \left( \frac{|\Delta {\varvec{X}}_{{\alpha }' {\alpha }}|}{r} \right) -1 + \frac{|\Delta {\varvec{X}}_{{\alpha }' {\alpha }}|}{r} \right] {+}\beta '_\mathrm{w} \frac{r}{2}\\&\quad \times \sum _{<{\alpha },\mathrm{wall}>} \left[ -\log \left( \frac{|\Delta {\varvec{X}}_{\mathrm{wall},{\alpha }}|}{r/2} \right) -1 + \frac{|\Delta {\varvec{X}}_{\mathrm{wall},{\alpha }}|}{r/2} \right] \end{aligned} \end{aligned}$$

with $\Delta {\varvec{X}}_{{\alpha }'{\alpha }}= {\varvec{X}}_{{\alpha }'}- {\varvec{X}}_{{\alpha }}$, where *r* is the cell–cell interaction range and roughly means the cell diameter. The summation $\sum _{<{\alpha },{\alpha }'>}$ runs for all the pairs of neighboring cells (${\alpha }$-th and ${\alpha }'$-th) satisfying $|\Delta {\varvec{X}}_{{\alpha }'{\alpha }}|<r$. The second term, cell-wall volume exclusion, is defined similarly but with $|\Delta {\varvec{X}}_{\mathrm{wall},{\alpha }}|<r/2$ for the interaction range.

Note that Eq. (2) can be rewritten as

5 $$\begin{aligned} \frac{\mathrm{d} {\varvec{X}}_{\alpha }}{\mathrm{d}t} = v_0 \hat{\varvec{q}}_{\alpha } + {\varvec{J}^{v}}_{\alpha } \end{aligned}$$

with the interaction term ${\varvec{J}^{v}}_{\alpha } = - \varGamma \partial E / \partial {\varvec{X}}_{\alpha }$ and the coefficient $v_0 \equiv \varGamma e$ giving the fixed speed of migration for the ${\varvec{J}^{v}}_{\alpha }=0$ case, or a migrating cell without any interaction. From Eq. (4), ${\varvec{J}^{v}}_{\alpha }$ is given by

6 $$\begin{aligned} \begin{aligned} {\varvec{J}^{v}}_{\alpha }&= - \beta \sum _{{\alpha }' \ (n. {\alpha })} \left( \frac{r}{ |\Delta {\varvec{X}}_{{\alpha }'{\alpha }}|} - 1 \right) \widehat{\varvec{\Delta }{\varvec{X}}_{{\alpha }'{\alpha }} } \\&\quad - \beta _\mathrm{w} \sum _{\mathrm{wall} \ (n. {\alpha })} \left( \frac{r/2}{ |\Delta {\varvec{X}}_{\mathrm{wall},{\alpha }}|} - 1 \right) \widehat{\varvec{\Delta }{\varvec{X}}_{\mathrm{wall},{\alpha }} } \\&\quad - \varGamma \frac{\partial E_\mathrm{A-B}}{\partial {\varvec{X}}_{\alpha }} \ , \end{aligned} \end{aligned}$$

where $\widehat{\varvec{\Delta }{\varvec{X}}_{{\alpha }'{\alpha }} }={\varvec{\Delta }{\varvec{X}}_{{\alpha }'{\alpha }} }/|{\varvec{\Delta }{\varvec{X}}_{{\alpha }'{\alpha }} }|$ and $\beta \equiv \varGamma \beta '$. The summation $\sum _{{\alpha }' \ (n. {\alpha })}$ runs for all the neighbors of ${\alpha }$-th cell satisfying $|\Delta {\varvec{X}}_{{\alpha }'{\alpha }}|<r$. This definition is also applied to $\sum _{\mathrm{wall} \ (n.{\alpha })}$ but with the range *r*/2.

Equation (3) describes the time evolution rule for the intrinsic polarity ${\varvec{q}}_{\alpha }$ of the ${\alpha }$-th particle, or at the basal side of ${\alpha }$-th cell. The first term of the right-hand side in Eq. (3) controls the spontaneous formation of the polarity of each particle, with the coefficient $I_q$ specifying the strength of polarity formation and the optimum magnitude *Q*. The second term ${\varvec{J}^{q}}_{\alpha }$ represents interaction between the particles through the intrinsic polarity, which is further specified below. The last term $\varvec{\xi }_{\alpha }(t)$ specifies the noise for the polarity; this noise is assumed to be a white Gaussian noise with $\langle {\varvec{\xi }}_{\alpha } \rangle = 0$ and

7 $$\begin{aligned} \langle \xi _{{\alpha },k}(t) \xi _{{\alpha }',l}(t') \rangle = 2 D \delta _{{\alpha }, {\alpha }'} \delta _{kl} \delta (t-t') \, \end{aligned}$$

where the subscripts *k* and *l* specify the directions, $k, l =x , y$. The coefficient *D* indicates the noise strength and has the unit of inverse time.

In this article, for polarity interaction ${\varvec{J}^{q}}_{\alpha }$ we adopt the following combination of contact communication through the basal sides of cells. Following Ref. [14], we assume that this term ${\varvec{J}^{q}}_{\alpha }$ is given by the additive combination of CF and CIL/CAL terms, *i.e.* ${\varvec{J}^{q}}_{\alpha } = {\varvec{J}^\mathrm{CF}}_{\alpha } + {\varvec{J}^\mathrm{CIL}}_{\alpha }$, and these two terms are defined as follows; ${\varvec{J}^\mathrm{CF}}_{\alpha }$ represents the CF contribution

8 $$\begin{aligned} {\varvec{J}^\mathrm{CF}}_{\alpha } = \alpha _\mathrm{CF} \sum _{{\alpha }' \ (n. {\alpha })} \frac{1 + \widehat{\varvec{\Delta }{\varvec{X}}_{{\alpha }'{\alpha }}} \cdot \widehat{\varvec{q}_{{\alpha }'}} }{2} \widehat{\varvec{\Delta }{\varvec{X}}_{{\alpha }'{\alpha }} } \end{aligned}$$

and ${\varvec{J}^\mathrm{CIL}}_{\alpha }$ represents the CIL/CAL contribution (for $\alpha _\mathrm{CIL}>0$/$<0$, resp.)

9 $$\begin{aligned} {\varvec{J}^\mathrm{CIL}}_{\alpha } = -\alpha _\mathrm{CIL} \sum _{{\alpha }' \ (n.{\alpha })} \left( \frac{r}{ |\Delta {\varvec{X}}_{{\alpha }'{\alpha }}|} - 1 \right) \widehat{\varvec{\Delta }{\varvec{X}}_{{\alpha }'{\alpha }} } . \end{aligned}$$

Again, the summation $\sum _{{\alpha }' \ (n.{\alpha })}$ runs for all neighbors of ${\alpha }$-th cell satisfying $|\Delta {\varvec{X}}_{{\alpha }'{\alpha }}|<r$. Here, $\alpha _\mathrm{CF}$ is the strength of contact follow (CF) and should be $\alpha _\mathrm{CF}\ge 0$, and $\alpha _\mathrm{CIL}$ is the strength of contact inhibition/attraction of locomotion (CIL/CAL). For CIL, $\alpha _\mathrm{CIL}>0$, whereas for CAL, $\alpha _\mathrm{CIL}<0$.

Here we briefly review how each of these terms affects the population dynamics of freely migrating cells, or self-propelled particles, described in our earlier work [14]. The CF term gives rise to a nonreciprocal local interaction with forward-backward asymmetry (Fig. 1b right). In terms of symmetry, this is similar to the chasing interaction assumed in the escape-and-pursuit and cognitive flocking models [33–36], and can induce, e.g., chain migration. Indeed, our previous work [14] reported the rotating rings and spirals and dynamic assemblies with snake-like stripe shapes due to this CF term. The CIL term gives rise to an effective short-range repulsion acting on the intrinsic polarity ${\varvec{q}}$ (Fig. 1b left) when the coefficient $\alpha _\mathrm{CIL}$ is positive. This is similar to local cognitive repulsion in the animal group model [34]. CIL combined with the volume exclusion effectively causes alignment due to the pair behavior analogous to inelastic collision [13, 37–39]. When the coefficient $\alpha _\mathrm{CIL}$ is negative, this CIL term works as CAL, which is here simply assumed as the inverse direction of CIL (Fig. 1b center), and gives rise to an effective short range attraction on ${\varvec{q}}$. Aggregation patterns with various dynamics were obtained due to CAL [14]. Furthermore, our model for the freely migrating case [14] exhibits polar traveling bands and homogeneous polar flocking for $\alpha _\mathrm{CIL} >0$ (repulsion), whereas aggregation for $\alpha _\mathrm{CIL} <0$ (attraction), which is similar to the collective motion due to short-range selective attraction–repulsion [40].

CIL is recognized as an important cell–cell type of communication for coherent cell migration and has been introduced in several earlier theoretical works [24, 41]. The definition of CIL given by Eq. (9) is similar to that of Ref. [24] except for the following details. Our definition has cell–cell distance dependency and is additive among the cells in contact, which are different from Ref. [24]. The definition of the cells in contact is also different; our definition is based on the distance between the centroids of the two cells at basal sides, whereas the definition in Ref. [24] is based on the shared vertex at apical sides. The model in Ref. [41] uses the theoretical framework which explicitly describes the deformable cell shape, and CIL is implemented by explicitly introducing the density distribution of polarity molecules and its corresponding dynamics.

In what follows, we assume $I_q$ is infinitely large, and hence the polarity amplitude $|{\varvec{q}}|$ is fixed and only its direction can vary (i.e., $I_q \rightarrow \infty$ and $|{\varvec{q}}_{\alpha }| = Q$). This allows us to replace Eq. (3) by the time evolution of the angles $\theta _j$ of the polarity directions, ${\varvec{q}}_{\alpha } = (Q \cos \theta _{\alpha }, Q \sin \theta _{\alpha })$. This replacement makes the numerical calculation stable since we can avoid the large coefficient $I_q$. Note that, since Eq. (5) does not depend on the magnitude of the polarity vector *Q*, we can rescale Eq. (3) and set $Q=1$ without loss of generality, just by redefining $\tilde{\varvec{q}}_{\alpha } = {\varvec{q}}_{\alpha }/Q$, $\tilde{I_q} = I_q Q^2$ and $\tilde{\varvec{\xi }^{q}}_{\alpha } ={\varvec{\xi }^{q}}_{\alpha }/Q$

and the coefficients in ${\varvec{J}^{q}}_{\alpha }$ accordingly. Together, Eq. (3) is reduced into

10 $$\begin{aligned} \frac{\mathrm{d} \theta _{\alpha }}{\mathrm{d}t} = - ({J_x^{q}}_{\alpha } + f_{x{\alpha }} ) \sin \theta _{\alpha } + ({J_y^{q}}_{\alpha } + f_{y{\alpha }} ) \cos \theta _{\alpha } + \xi _{\alpha } \ . \end{aligned}$$

(For the brevity of notations, the tilde $\tilde{\cdot }$ on each variable has been omitted.) Here, $\xi _{\alpha }$ is the angular noise defined by $\xi _{\alpha } = - \xi ^{q}_{x,{\alpha }} \sin \theta _{\alpha } + \xi ^{q}_{y,{\alpha }} \cos \theta _{\alpha }$, which satisfies $\langle \xi _{\alpha }(t) \rangle =0$ and

11 $$\begin{aligned} \langle \xi _{\alpha }(t) \xi _{\alpha '} (t') \rangle = 2 D \delta _{{\alpha },{\alpha }'} \delta (t-t') . \end{aligned}$$

Note that $D^{-1}$ now characterizes the persistence time of polarity direction.

### Model for apical constraint: Vertex model

For this part, we apply the cellular vertex model in two dimensions (*x*, *y*) [18, 42–48]. We describe the cell shapes by the 2D polygons as mentioned above, which is specified by the locations of vertices $\varvec{r}_i=(r^x_i,r^y_i)$ (for *i*-th vertex; $i=1,2, \ldots , M$) and their connectivity $\langle k l \rangle$ representing the edge which connects vertexes *k* and *l*. The time evolution of each vertex is given by

12 $$\begin{aligned} \frac{\mathrm{d}{ \varvec{r}_i }}{\mathrm{d}t}= - \kappa \frac{\partial E(\{ \varvec{r}_i \})}{\partial { \varvec{r}_i }} \end{aligned}$$

with the mobility coefficient $\kappa$.

In the potential *E*, the parts relevant for the apical side dynamics are those defined within the apical side $E_\mathrm{vertex}$ and for the apico-basal connection $E_\mathrm{A-B}$ (Fig. 1c). (For $E_\mathrm{A-B}$, see Sect. 2.3.) The pure apical contribution $E_\mathrm{vertex}$ is given by four different sub-parts as

13 $$\begin{aligned} E_\mathrm{vertex} = E_\mathrm{pres} + E_\mathrm{tens} + E_\mathrm{fluc} + E_\mathrm{wall} \ \end{aligned}$$

and Eq. (12) is now written as

14 $$\begin{aligned} \begin{aligned} \frac{\mathrm{d}{ \varvec{r}_i }}{\mathrm{d}t}&= - {\kappa }_{i} \left[ \frac{\partial E_\mathrm{pres}}{\partial { \varvec{r}_i }} + \frac{\partial E_\mathrm{tens}}{\partial { \varvec{r}_i }} + \frac{\partial E_\mathrm{fluc}}{\partial { \varvec{r}_i }} \right. \\&\qquad \qquad \left. + \frac{\partial E_\mathrm{wall}}{\partial { \varvec{r}_i }} + \frac{\partial E_\mathrm{A-B}}{\partial { \varvec{r}_i }} \right] \ . \end{aligned} \end{aligned}$$

The first term of Eq. (13), $E_\mathrm{pres}$, represents a weak constraint giving optimum in-plane area of each cell. This is given by

15 $$\begin{aligned} E_\mathrm{pres} = \frac{K_a}{2}\sum \limits _{\alpha =1}^{N} ({{A}_{\alpha }}/{{A}_{0}}-1)^2 \end{aligned}$$

with the actual area $A_\alpha$ of the $\alpha$th cell, the preferred area $A_0$, and the coefficient $K_a$ controlling the strength of the pressure. The area $A_\alpha$ is calculated by the formula $A_\alpha = \sum _{ j \in \alpha -\mathrm {cell}} (r^x_j r^y_{j+1} - r^y_j r^x_{j+1})/2$, where the summation $\sum _{ j \in \alpha -\mathrm {cell}}$ runs for all the vertices *i* (numbered in the counter-clockwise order) belonging to $\alpha$-th cell. The second term $E_\mathrm{tens}$ represents the potential constraining the total perimeter on cell–cell junction, given by

16 $$\begin{aligned} E_\mathrm{tens} = \frac{K_p}{2}\sum \limits _{\alpha =1}^{N}{(L_{\alpha }/L_0-1)^2} \end{aligned}$$

with the actual and optimum perimeters of the $\alpha$-th cell $L_\alpha$ and $L_0$, respectively, and the coefficient $K_p$ controlling the tension strength. The third term $E_\mathrm{fluc}$ is introduced to implement the fluctuation in line tension in each cell–cell junction, for which we assume

17 $$\begin{aligned} E_\mathrm{fluc} = \sum \limits _{\langle ij \rangle } \gamma _{ij} (t) \ell _{ij} \ . \end{aligned}$$

Here, $\ell _{ij}$ is the length of the bond connecting vertices *i* and *j*, $\ell _{ij} \equiv |{\varvec{r}}_i - {\varvec{r}}_j|$. The time-evolution of junction-specific tension $\gamma _{ij}$ is assumed to obey

18 $$\begin{aligned} \frac{\mathrm{d} \gamma _{i j}(t)}{\mathrm{d}t}=-\frac{1}{\tau } \gamma _{i j}(t) + \eta _{ij}(t) \ , \end{aligned}$$

where $\eta _{ij}(t)$ is a white Gaussian noise satisfying $\langle \eta _{ij}(t) \rangle =0$ and $\langle \eta _{ij}(t_1) \eta _{kl}(t)(t_2) \rangle = \sigma ^2 \delta _{ik}\delta _{jl}\delta (t_1-t_2)$. The parameters $\tau$ and $\sigma$ represent the relaxation time and strength of noise, respectively. The last term $E_\mathrm{wall}$ is the potential to implement the boundaries of the system, for which we assume

19 $$\begin{aligned} \begin{aligned} E_\mathrm{wall}&= \frac{k_\mathrm{wall, 1}}{2} \left[ \sum _{i\in \mathcal {X+}} ({r^x}_i - X_{+})^2 + \sum _{i\in \mathcal {X-}} ({r^x}_i - X_{-})^2 \right. \\&\quad + \left. \sum _{i\in \mathcal {Y+}} ({r^y}_i - Y_{+})^2 + \sum _{i\in \mathcal {Y-}} ({r^y}_i - Y_{-})^2 \right] \\&\quad + \frac{k_\mathrm{wall, 2}}{2} \left[ \sum _{i: \ {r^x}_i>X_{+}} ({r^x}_i - X_{+})^2 \right. \\&\quad + \sum _{i: \ {r^x}_i<X_{-}} ({r^x}_i - X_{-})^2 \\&\quad + \left. \sum _{i: \ {r^y}_i>Y_{+}} ({r^y}_i - Y_{+})^2 + \sum _{i: \ {r^y}_i<Y_{-}} ({r^y}_i - Y_{-})^2 \right] \ . \end{aligned} \end{aligned}$$

Here, $X_{+}$ and $X_{-}$ are the locations of the walls along *x*-axis, whereas $Y_{+}$ and $Y_{-}$ are the locations of the walls along *y*-axis. $k_\mathrm{wall, 1}$ and $k_\mathrm{wall, 2}$ are the coefficients specifying the strength of the confinement by the wall. (In general, the coefficients $k_\mathrm{wall, 1}$ and $k_\mathrm{wall, 2}$ can be different with each other, but later we assume the same value for both. The choices of these values may not cause anything significant as long as the values are enough large.) The summations $\sum _{i\in \mathcal {X+}}$ and $\sum _{i\in \mathcal {X-}}$ run over all vertices which locate at ${r^x}_i>X_{+}-\delta$ and ${r^x}_i<X_{-}+\delta$, respectively, and belong to only two cells. The summations $\sum _{i\in \mathcal {Y+/-}}$ are defined similarly. The parameter $\delta$ is introduced to specify the cells located near the system boundary. In this model, with positive $\delta$, the vertices at the boundary adhere to the boundary wall.

Cell rearrangement is implemented by junctional remodeling (JR) in the standard way. When a bond becomes shorter than a threshold length $\epsilon$ during the time evolution of vertex locations, the bond is rotated by 90 degrees around its midpoint. Simultaneously, the five edges connected to either of two vertices at the JR site are reconnected such that the T1 transition is achieved; see *e.g.* Refs. [18, 46, 49].

### Connection between apical and basal sides

We naively assume that the basal side (self-propelled particle) of the ${\alpha }$-th cell is connected to the vertices at the apical side of the same cell by a linear elastic spring. The spring is represented by the following potential function

20 $$\begin{aligned} E_\mathrm{A-B}(\{ {\varvec{r}}_i \}, \{ {\varvec{X}}_{\alpha } \} ) = \frac{K}{2} \sum _{\alpha } \left[ {\varvec{R}}_{\alpha } (\{ {\varvec{r}}_i \}) - {\varvec{X}}_{\alpha } \right] ^2 \end{aligned}$$

where the coefficient *K* means a spring constant, ${\varvec{R}}_{\alpha }$ represents the centroid of the vertices of the $\alpha$-th cell ${\varvec{R}}_{\alpha }= [1/(6A_{\alpha })] \sum _{ i \in \alpha -\mathrm {cell}} ( ({r^x}_i+{r^x}_{i+1}) ({r^x}_i {r^y}_{i+1} - {r^x}_{i+1} {r^y}_{i}), ({r^y}_i+{r^y}_{i+1}) ({r^x}_i {r^y}_{i+1} - {r^x}_{i+1} {r^y}_{i}) )$, and the summation $\sum _{ i \in \alpha -\mathrm {cell}}$ runs for all the vertices *i* (numbered in the counter-clockwise order) belonging to $\alpha$-th cell.

## Results

**Fig. 2:**
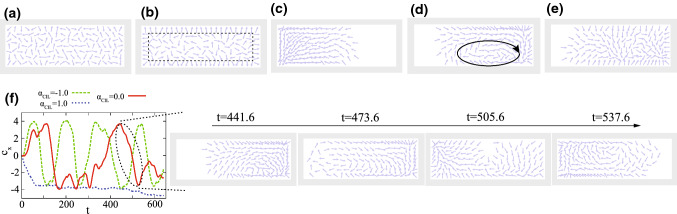
Simulation results for freely migrating cells under spatial confinement. **a**–**e** Snapshots for **a** $(\alpha _\mathrm{CIL},\alpha _\mathrm{CF})=(0.0, 0.0)$, **b** $(\alpha _\mathrm{CIL},\alpha _\mathrm{CF})=(2.0, 0.0)$, **c** $(\alpha _\mathrm{CIL},\alpha _\mathrm{CF})=(1.0, 1.0)$, **d** $(\alpha _\mathrm{CIL},\alpha _\mathrm{CF})=(0.0, 1.0)$ and **e** $(\alpha _\mathrm{CIL},\alpha _\mathrm{CF})=(-2.0, 0.5)$. **f** Time evolution in *x*-position of the centroid of all cells $c_x$ for $\alpha _\mathrm{CF}=1.0$ and various $\alpha _\mathrm{CIL}$ ($=-1.0$, 0.0 and 1.0) and the time series of the snapshots corresponding to (**d**)

In this section, we present the results obtained by numerical simulations of the model presented in Sect. 2 [Eqs.  (5), (10) and (14) with Eqs. (6), (8), (9), (11), (15), (16), (17), (18), (19) and (20)]. The parameter values which we use for the simulations in this article are as follows. Cell–cell communication strengths $\alpha _\mathrm{CF}$ and $\alpha _\mathrm{CIL}$ are the control parameters in this article and are varied over $0.0 \le \alpha _\mathrm{CF} \le 1.0$ and $-2.0 \le \alpha _\mathrm{CIL} \le 2.0$. The spring coefficient of the apico-basal connection *K* is another control parameter, which is varied within the range from $1.0 \times K_0$ to $6.0 \times K_0$ with a certain $K_0$ defined right below to set the force unit. The shape factor $P \equiv L_0/\sqrt{A_0}$ is the other control parameter and is varied around the so-called solid-to-fluid transition threshold ($P\sim 3.812$) [52]; $3.700 \le P \le 3.880$. We set the units of length, time and force by $A_0$, $\sqrt{A_0}/v_0$ and $K_0 \sqrt{A_0}$, respectively, and hence, in the equations, always put $A_0=1$, $v_0=1$ and $K_0=1$ (i.e., when $K=1.0$, the migration driving force of a single cell has ability to displace the centroid of the basal side from that of the apical side over the unit length, which is roughly the diameter of a cell). The total number of cells is $N=140$. The system size is $2 X_{+}=-2 X_{-}=(20.0/7.0)^{1/2} \sqrt{N A_0}$ and $2 Y_{+}=-2 Y_{-}=(20.0/7.0)^{-1/2} \sqrt{N A_0}$ along *x* and *y*, respectively. (Only in “Appendix A”, the system size is $\sqrt{N A_0}$ along both *x* and *y*.) The energy scales of the vertex model are assumed enough large, as $K_a=150.0$ and $K_p=150.0$, which sets the relaxation time scale of the apical side dynamics to be much smaller than the unit time. The other parameters are given as follows unless otherwise mentioned: $\beta =5.0$, $\beta _\mathrm{w}=100.0$, $r=(1+v_0/\beta ) r_0$ with $r_0 = 2.0 \sqrt{A_0/\pi }$ (*r* corresponds to the diameter of a cell at the basal side, whereas $r_0$ roughly corresponds to a typical cell diameter at the apical side, respectively), $D=0.1 \times (r/v_0)$, $\kappa = 1.0$, $\sigma =0.0$ except for “Appendix B”, where $\sigma =0.5$, $\tau = r_0/v_0$, $\epsilon =0.05 \times r_0$, $k_\mathrm{wall, 1}=100.0$, and $k_\mathrm{wall, 2}=100.0$. For the ensemble average, the number of realizations for each parameter value set is 128, 64, 24 and 16 for Figs. 4a, c and 9a, c, Fig. 6c, Figs. 5, 6a, b and 10 and the others, respectively. In this model, perhaps due to non-variation dynamics nature by the self-propulsion term, sometimes the illegal process of cellular vertex dynamics occurred; namely, a vertex gets across some cell–cell junction, and caused the simulation failure. For example, for $(\alpha _\mathrm{CF},\alpha _\mathrm{CIL},P)=(0.0,0.0,3.880)$, we had no failures out of 128 realizations, whereas for the most dangerous case, $(\alpha _\mathrm{CF},\alpha _\mathrm{CIL}, P)=(1.0,0.0,3.880)$, we had 43 failures out of 128 realizations. The realizations which caused such failure were excluded from the ensemble average.

For the numerical integration, time is discretized with the increment $dt=0.0001$ and $dt=0.001$ for Fig. 7 and the other results, respectively. The maximum number of steps for each simulation run is 640, 000 (in terms of time, $t=640$), 1, 600, 000 ($t=160$) and 160, 000 ($t=160$) for Figs. 2f, 7 and the other results, respectively.

### Freely migrating cells under spatial confinement

We start with studying the case when the apical constraint is absent so that the cells can freely migrate. In other words, this is the limit when the apical side dynamics is perfectly dominated by the basal side dynamics and the apical restriction does not affect the basal side dynamics at all. Figure 2a–e shows the snapshots of the numerical simulations for $(\alpha _\mathrm{CIL},\alpha _\mathrm{CF})=(0.0,0.0)$, $(\alpha _\mathrm{CIL},\alpha _\mathrm{CF})=(2.0,0.0)$, $(\alpha _\mathrm{CIL},\alpha _\mathrm{CF})=(1.0,1.0)$, $(\alpha _\mathrm{CIL},\alpha _\mathrm{CF})=(0.0,1.0)$ and $(\alpha _\mathrm{CIL},\alpha _\mathrm{CF})=(-2.0, 0.5)$. In the absence of any cell–cell communication, or for $(\alpha _\mathrm{CIL},\alpha _\mathrm{CF})=(0.0, 0.0)$, cells are apparently well distributed (Fig. 2a). In other words, we are using the values of parameter $v_0$ and the number density of the particles which do not induce motility-induced phase separation [50, 51]. For $(\alpha _\mathrm{CIL},\alpha _\mathrm{CF})=(2.0,0.0)$, the cells form the dense shell-like structure near the wall, as indicated by the broken line, which provides the inner cells with more spaces and hence the inner cells can become more motile (Fig. 2b). In contrast, in the presence of enough strong contact follow, *e.g.* for $(\alpha _\mathrm{CIL},\alpha _\mathrm{CF})=(1.0, 1.0)$, the cells accumulate in the half side of the system, which suppress the cells’ motility (Fig. 2c). Even with such large $\alpha _\mathrm{CF}$, when the $\alpha _\mathrm{CIL}$ is enough close to zero or negative, e.g., $(\alpha _\mathrm{CIL},\alpha _\mathrm{CF})=(0.0, 1.0)$, the coherent swirling motion is observed in the cell accumulation (Fig. 2d). In this swirling regime, cells have much higher motility than the three regimes mentioned above. (Therefore, in what follows, we call this regime/state highly-motile regime/state.) Furthermore, when we decrease $\alpha _\mathrm{CF}$ from this regime, we see the situation where swirling is not obvious but still stream line is visible (Fig. 2e).

**Fig. 3:**
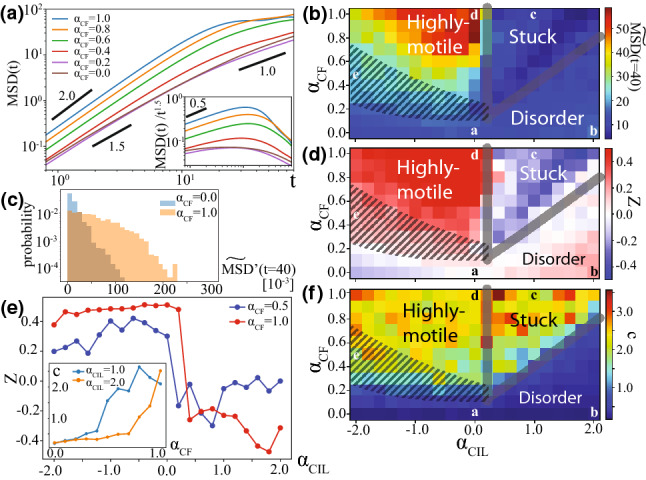
Simulation results for freely migrating cells under spatial confinement. **a** Mean square displacement (MSD) for $\alpha _\mathrm{CIL}=0.0$ and various $\alpha _\mathrm{CF}$. Inset: MSD divided by $t^{1.5}$. **b** $\widetilde{\mathrm{MSD}}(t=40)$ against $\alpha _\mathrm{CIL}$ and $\alpha _\mathrm{CF}$. **c** Histogram of $\widetilde{\mathrm{MSD}}'(t=40)$ for $\alpha _\mathrm{CF}=0.0$ and 1.0 with $\alpha _\mathrm{CIL}=0.0$. **d** *Z* value, defined in Eq. 22 against $\alpha _\mathrm{CIL}$ and $\alpha _\mathrm{CF}$. **e** *Z* value as a function of $\alpha _\mathrm{CIL}$ for $\alpha _\mathrm{CF}=0.5$ and 1.0. Inset: Average distance *c* between the centroid of cells and the center of the system (0, 0) as a function of $\alpha _\mathrm{CF}$ for $\alpha _\mathrm{CIL}=1.0$ and 2.0. **f** Average distance *c* between the centroid of cells and the center of the system (0, 0) against $\alpha _\mathrm{CIL}$ and $\alpha _\mathrm{CF}$. In (**b**, **d**, **f**), the alphabets indicate the parameter values used for the snapshots in Fig. 2a–e

In the highly-motile regime, the side in which cells are accumulated switches time-by-time, as shown by the time-evolution of *x*-component $c_x (t)$ of the centroid among all cells with ${\varvec{c}}(t) =(1/N) \sum _{{\alpha } = 1}^{N} (X_{\alpha }(t), Y_{\alpha }(t))$ (Fig. 2f graph red solid curve and snapshots). For $\alpha _\mathrm{CF}=1.0$, this switching seems like the regular oscillation when the $\alpha _\mathrm{CIL}$ is negative and the absolute value is enough large (Fig. 2f graph green broken curve; $\alpha _\mathrm{CIL}= -1.0$), whereas it seems to occur in a stochastic manner near $\alpha _\mathrm{CIL}=0$ (Fig. 2f graph red solid curve; $\alpha _\mathrm{CIL}= 0.0$). For further increased $\alpha _\mathrm{CIL}$ (i.e., $\alpha _\mathrm{CIL}> 0.0$), the switching does not occur (Fig. 2f graph blue dotted curve; $\alpha _\mathrm{CIL}= +1.0$)

We now focus on the motility of cells depending on the cell–cell communication parameters. Due to the spatial confinement and cell–cell volume exclusion, cells in the current situation are basically much less motile than a single freely migrating cell without any constraint. We will see how and when the motility is improved back associated with the aforementioned coherent dynamics induced by cell–cell communication.

We measure the mean square displacement of each cell (${\alpha }$-th cell)

21 $$\begin{aligned} \mathrm{MSD'}_j(t) = \frac{1}{T_\mathrm{end} - T_\mathrm{ini}} \int _{T_\mathrm{ini}}^{T_\mathrm{end}} dt_0 \left| \int _{0}^{t}dt' {\varvec{v}}_{\alpha }(t_0 + t) \right| ^2 \ . \end{aligned}$$

The MSD, defined over all the cells, is calculated by $\mathrm{MSD}(t) = \langle (1/N) \sum _{{\alpha } = 1}^{N} \mathrm{MSD'}_{\alpha }(t) \rangle$, where $\langle \cdot \rangle$ represents the ensemble average. Figure 3a shows the MSD against time difference *t* for various $\alpha _\mathrm{CF}$ for $\alpha _\mathrm{CIL}=0.0$. For $\alpha _\mathrm{CF}=0.0-0.6$, the crossover from super-diffusive to normal diffusion appears at ca. $t = 10$ to 20. (For $\alpha _\mathrm{CF}=0.8$ and 1.0, the saturation due to the finite system size occurs.) This is consistent with our parameter setting $D=0.1$ because the persistence time of motile direction is given by $\sim 1/D \sim 10.0$. Therefore, values of MSD at $t=40$, normal diffusion regime are picked up and plotted in 3b for various $\alpha _\mathrm{CIL}$ and $\alpha _\mathrm{CF}$. Hereafter, when MSD at the fixed time difference *t* is plotted, we use the rescaled one $\widetilde{\mathrm{MSD}}(t) = \mathrm{MSD}(t)/(4D_0 t)$, where $D_0$ means the effective diffusion constant of a single cell, $D_0 \equiv v_0^2/(2D)$, and $4D_0 t$ is its MSD [21]. ($D_0 \sim 6.75$ for the current setting.)

In the above analysis, we averaged MSD over all the cells. However, in the highly motile regime of this simulation, only part of the cells might migrate dominantly. Cells in the region where swirling takes place can have larger MSD values than those far from such swirling region. This fact is indeed captured by the histogram of each cell MSD shown in 3c, where, for $\alpha _\mathrm{CIL}=0.0$, $\mathrm{MSD'}_{\alpha }(t=40)$ for $\alpha _\mathrm{CF}=0.0$ drops exponentially, whereas $\mathrm{MSD'}_{\alpha }(t=40)$ for $\alpha _\mathrm{CF}=1.0$ has slowly decaying tail. Therefore, to focus on only the cells which are moving largely, we pick up the top $10\%$ of $\mathrm{MSD'}_{\alpha }(t=40)$ over all the cells and samples for each parameter set $(\alpha _\mathrm{CIL},\alpha _\mathrm{CF})$ and calculate their average by $\mathrm{MSD}^{10\%,t=40}_{(\alpha _\mathrm{CIL},\alpha _\mathrm{CF})}$. Figure 3d plots

22 $$\begin{aligned} Z \equiv \frac{ \mathrm{MSD}^{10\%,t=40}_{(\alpha _\mathrm{CIL},\alpha _\mathrm{CF})}-\mathrm{MSD}^{10\%,t=40}_{(0,0)} }{ \mathrm{MSD}^{10\%,t=40}_{(\alpha _\mathrm{CIL},\alpha _\mathrm{CF})}+\mathrm{MSD}^{10\%,t=40}_{(0,0)}} \ , \end{aligned}$$

which is the rescaled $\mathrm{MSD}^{10\%,t=40}_{(\alpha _\mathrm{CIL},\alpha _\mathrm{CF})}$ such that it gives 0 and 1 ($-1$) for $\mathrm{MSD}^{10\%,t=40}_{(\alpha _\mathrm{CIL},\alpha _\mathrm{CF})} / \mathrm{MSD}^{10\%,t=40}_{(0,0)} =1$ and $\gg 1$ ($\ll 1$), respectively. Here, the solid curve near $\alpha _\mathrm{CIL}=0.2$ in Fig. 3b, d shows the state boundaries of highly-motile and stuck regimes. The other state boundary appears at the upper edge of the region highlighted by the gray hatching.

These state boundaries surrounding the highly motile regime are determined as follows. The boundary near $\alpha _\mathrm{CIL} \sim 0$ is identified by the positive to negative jump of the *Z* value for increasing $\alpha _\mathrm{CIL}$, as shown in Fig. 3e. To determine the other boundary, there is still an ambiguity. It may be determined as the line where the *Z* value suddenly drops from  0.4 to  0.2 in Fig. 3e for decreasing $\alpha _\mathrm{CIL}$. However, it is difficult to identify whether the regime right below this boundary, including the case shown in Fig. 2e, is genuinely different from highly motile or disorder regime, and this is why we indicate such regime by the gray hatching in Fig. 3d. Note that the “streaming state” boundaries for the periodic boundary case given in “Appendix A,” determined by the analysis of the apparent global polar order, match these boundaries. This fact supports our definitions of the state boundaries.

The other state boundary of the stuck regime is identified by the degree of asymmetric cell distribution $c=\langle [ 1/(T_\mathrm{end} - T_\mathrm{ini}) ] \int _{T_\mathrm{ini}}^{T_\mathrm{end}} dt | {\varvec{c}}(t)| \rangle$ shown in Fig. 3f. See also Fig. 3e inset.

(More comprehensive discussions about the way the state boundaries are determined are given in “Appendix C”.)

### Epithelial cells under spatial confinement

**Fig. 4:**
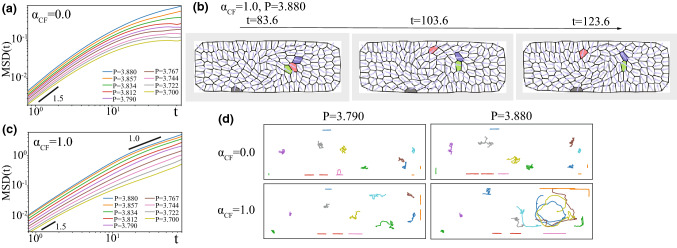
Simulation results for migrating cells under spatial confinement and epithelial integrity for $\alpha _\mathrm{CIL}=0.0$. **a** Mean square displacement for various shape index *P* for $\alpha _\mathrm{CF}=0.0$ and $\alpha _\mathrm{CIL}=0.0$. **b** Snapshots for $P=3.880$, $\alpha _\mathrm{CF}=1.0$ and $\alpha _\mathrm{CIL}=0.0$. **c** Mean square displacement for various shape index *P* for $\alpha _\mathrm{CF}=1.0$ and $\alpha _\mathrm{CIL}=0.0$. **d** Trajectories of 14 cells (position at the basal side) from $t=80$ to 160 for various parameters; $P=3.790$ and 3.880, and $\alpha _\mathrm{CF}=0.0$ and 1.0. $K=4.0$

We here investigate the case with apical constraint. We set $K=4.0$, which is larger than the unit 1.0 by several factors so that the discrepancy between the centroid positions of the basal and apical sides of each cell can be ignored. To check the consistency with literature, we firstly analyze the MSD in the absence of any cell–cell communication. The result is shown in Fig. 4a for various shape index *P*. At $t \gg 10$, the plateau is observed for $P \le 3.812$, whereas the diffusive behavior appears for $P>3.812$. This result is consistent with the solid-to-fluid transition in early literature [52] (for small driving force limit in their result). Note that this transition shape index $P=3.812$ matches those for the self-propelled Voronoi model at the small driving force limit [21]. This result may reflect the fact that the energy scales for apical constraints used in this article are so large ($K_a=K_p=150$) that the spontaneous motility at the basal side does not affect the dynamics at the apical side well without coherent dynamics among cells.

Next, we study the case with cell–cell communication at the basal side. We firstly focus on the case with only contact follow $\alpha _\mathrm{CF}$. Figure 4b shows the snapshots when the contact follow is incorporated, as $(\alpha _\mathrm{CIL},\alpha _\mathrm{CF})=(0.0, 1.0)$. As same as the free cell’s case (Fig. 2d), coherent swirling dynamics is observed. Figure 4c plots the MSD curves for $\alpha _\mathrm{CF}=1.0$ for various shape index *P*. In this case, even when the shape factor *P* is smaller than the transition threshold 3.812, the MSD curve exhibits the diffusion-like tendency at $t \gg 10$. These tendencies are also captured in the typical trajectories of cells shown in Fig. 4d. For $\alpha _\mathrm{CF}=1.0$, cells can still swirl under the apical constraint. Even in the solid regime, trajectories of some cells show large displacements in the presence of contact follow (e.g., $P=3.790$ and $\alpha _\mathrm{CF}=1.0$; bottom-left sub-figure in Fig. 4d). This result suggests that dynamic self-organization by contact follow communication can provide cells with collective driving force enough strong to overcome the apical constraints. Remember that we here set the vertex energy scales so large, or saying in another way, the self-propelled driving force is so weak, that migration ability of cells in the absence of intercellular communication does not affect the solid-to-fluid transition threshold *P*.

**Fig. 5:**
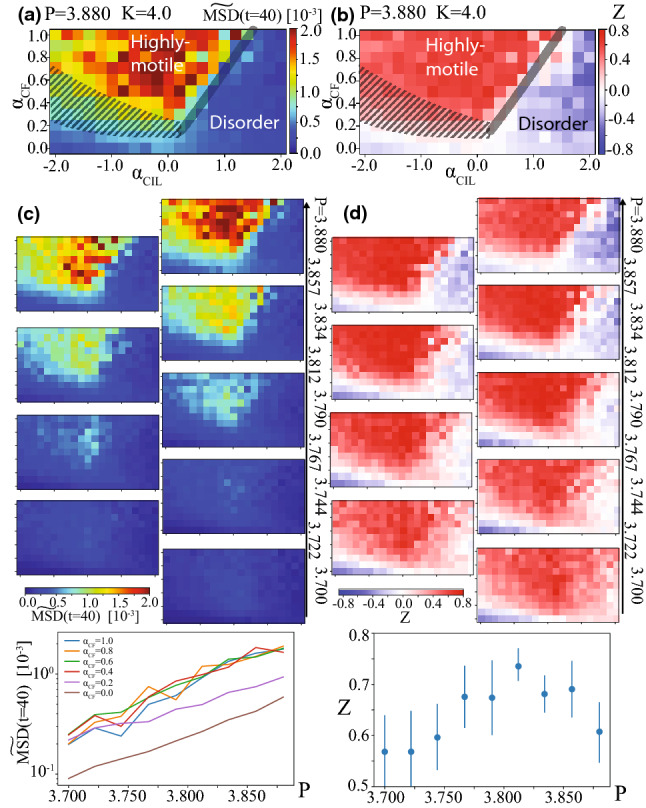
Simulation results for migrating cells under spatial confinement and epithelial integrity. **a** $\widetilde{\mathrm{MSD}}(t=40)$ against $\alpha _\mathrm{CIL}$ and $\alpha _\mathrm{CF}$ for the shape index $P=3.880$. **b** *Z* value, defined in Eq. 22 against $\alpha _\mathrm{CIL}$ and $\alpha _\mathrm{CF}$ for $P=3.880$. **c** $\widetilde{\mathrm{MSD}}(t=40)$ against $\alpha _\mathrm{CIL}$ and $\alpha _\mathrm{CF}$ for various *P*. The bottom graph plots $\widetilde{\mathrm{MSD}}(t=40)$ as a function of *P* for $\alpha _\mathrm{CIL}=0.0$ and various $\alpha _\mathrm{CF}$. **d** *Z* value, defined in Eq. 22 against $\alpha _\mathrm{CIL}$ and $\alpha _\mathrm{CF}$ for various *P*. The bottom graph plots *Z* value as a function of *P*; averaged among results for various $(\alpha _\mathrm{CIL}, \alpha _\mathrm{CF})$ for better statistics (within the range of $-0.4<\alpha _\mathrm{CIL}<0.4$ and $0.8< \alpha _\mathrm{CF}<1.0$). $K=4.0$. Error bars indicate standard deviations

We next perform numerical simulations for a various set of $\alpha _\mathrm{CF}$ and $\alpha _\mathrm{CIL}$. The dependence of MSD at $t=40.0$ on both $\alpha _\mathrm{CF}$ and $\alpha _\mathrm{CIL}$ is shown in Fig. 5a for a fluid phase, $P=3.880$. Although the tendency of the color map is similar to that in the free cell’s case (Fig. 3b), the highly motile regime is extended to the positive-$\alpha _\mathrm{CIL}$ side, e.g., up to $\alpha _\mathrm{CIL} \sim 1.0$ for $\alpha _\mathrm{CF}=1.0$. We also plot *Z* defined by Eq. 22 in Fig. 5b (for $P=3.880$). The parameter windows where cells are highly motile follows the similar trends with the free case, but it is extended to higher $\alpha _\mathrm{CIL}$ region as mentioned above. Compare the location of the highly motile regime boundary for this case ($\alpha _\mathrm{CIL}>0.0$; see Fig. 5a, b) with the corresponding one for the free case ($\alpha _\mathrm{CIL} \sim 0.0$; see Fig. 3b, d, f). This may be because the local cell density cannot be too high due to the apical area constraint. Note that this reasoning came from the following arguments. In our results, CIL prevents the swirling dynamics as we saw for freely migrating cells (Fig. 3b, d, f). (This may further stem from the CIL-induced effective alignment and the resultant emergence of the flocking state [13, 14, 37–39]. See “Appendix A” and Ref. [14] for more details about this.) This physical fact, together with the local cell density dependence of effective CIL strength, may be the reason of this enhancement of highly motile regime due to the apical area constraint. Although such density-dependence of effective CIL strength may be caused by the distance-dependent CIL coefficient in Eq. (9) in our case, the same result is expected as long as CIL strength effectively has the local density dependence for some mechanisms. It may be an interesting future direction to study how robust is this result for various assumptions for CIL.

**Fig. 6:**
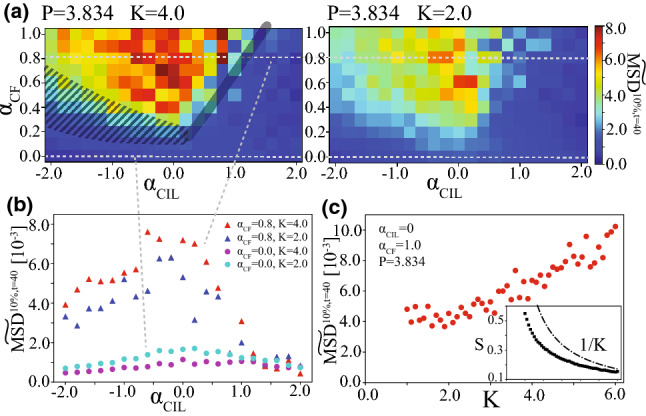
Dependencies on apico-basal connection strength *K*. For all figures here, we set $P=3.834$. **a** $\widetilde{\mathrm{MSD}}(t=40)$ of the top 10 percent against $\alpha _\mathrm{CIL}$ and $\alpha _\mathrm{CF}$ for $K=4.0$ and 2.0. Specifically, the cases for $\alpha _\mathrm{CF}=0.0$ and 0.8 are plotted in (**b**). **c** $\widetilde{\mathrm{MSD}}(t=40)$ of the top 10 percent against *K* for $(\alpha _\mathrm{CIL}, \alpha _\mathrm{CF})=(0.0, 1.0)$. Inset: Average distance between the centers of the apical and basal sides of each cell, *S*, against *K*. As naturally expected, the tendency roughly follows the 1/*K* curve. $P=3.834$

Figure 5c, d shows the maps of $\mathrm{MSD}(t=40)$ and *Z*-values for various *P*. As shown in Fig. 5c top, decreasing *P* just reduces the MSD value almost homogeneously over $(\alpha _\mathrm{CIL}, \alpha _\mathrm{CF})$. The color maps of *Z*-value for various *P* given in Fig. 5d shows that the optimum *P* exists. It is around the solid-fluid transition point, $P=3.812$. This optimality may appear because even the coherent dynamics is hindered for decreasing *P* when $P<3.812$ (the solid regime), whereas the base motility in the absence of cell–cell communication becomes larger for increasing *P* when $P>3.812$ (the fluid regime).

We performed the similar analysis for the case with the junctional tension fluctuation $\sigma =0.5$, as shown in “Appendix B”. In this case, the transition *P* becomes smaller (around $P\sim 3.790$), and indeed the optimum *P* for the *Z* value becomes smaller ($P\sim 3.790$) as well. “Appendix B” provides more results for the case with junctional tension fluctuation. Note also that we could not find any qualitative change of the results by this introduction of junctional tension fluctuation, besides the shift of transition *P* threshold.

Furthermore, in this mathematical model, we can tune the strength *K* of the elastic connection between apical and basal sides of each cell. Such *K*-dependency is briefly investigated in Fig. 6. Figure 6a, b compares $\mathrm{MSD}^{10\%,t=40}$ for $K=2.0$ and 4.0. In the highly motile regime, $\mathrm{MSD}^{10\%,t=40}$ for $K=4.0$ is higher than that for $K=2.0$. This is a striking contrast to the tendency in the disorder regime with lower $\alpha _\mathrm{CF}$, where the $\mathrm{MSD}^{10\%,t=40}$ for $K=4.0$ is lower than that for $K=2.0$, as shown in Fig. 6a, b, which is indeed natural because the basal dynamics is less restricted. Such characteristic *K*-dependency in the highly-motile regime is explicitly plotted in Fig. 6c. For $K>2.0$, $\mathrm{MSD}^{10\%,t=40}$ rises for increasing *K*.

For further smaller *K*, i.e., $K<2.0$, $\mathrm{MSD}^{10\%,t=40}$ seems to slightly increase for decreasing *K*, but this may be just because the basal side of a cell can move more easily around the centroid of its apical side. Indeed, the average distance *S* between the centroids of the apical and basal sides of each cell is plotted in Fig. 6c inset, showing the monotonic increase for decreasing elasticity *K*.

Here is a comment on the magnitude of *S* in this simulation. Within the range of *K* used in this simulation, *S* is near or less than 0.5 on average. This guarantees the validity of the model assuming a “phantom” spring between apical and basal sides.

### Epithelial cells in a free space

**Fig. 7:**
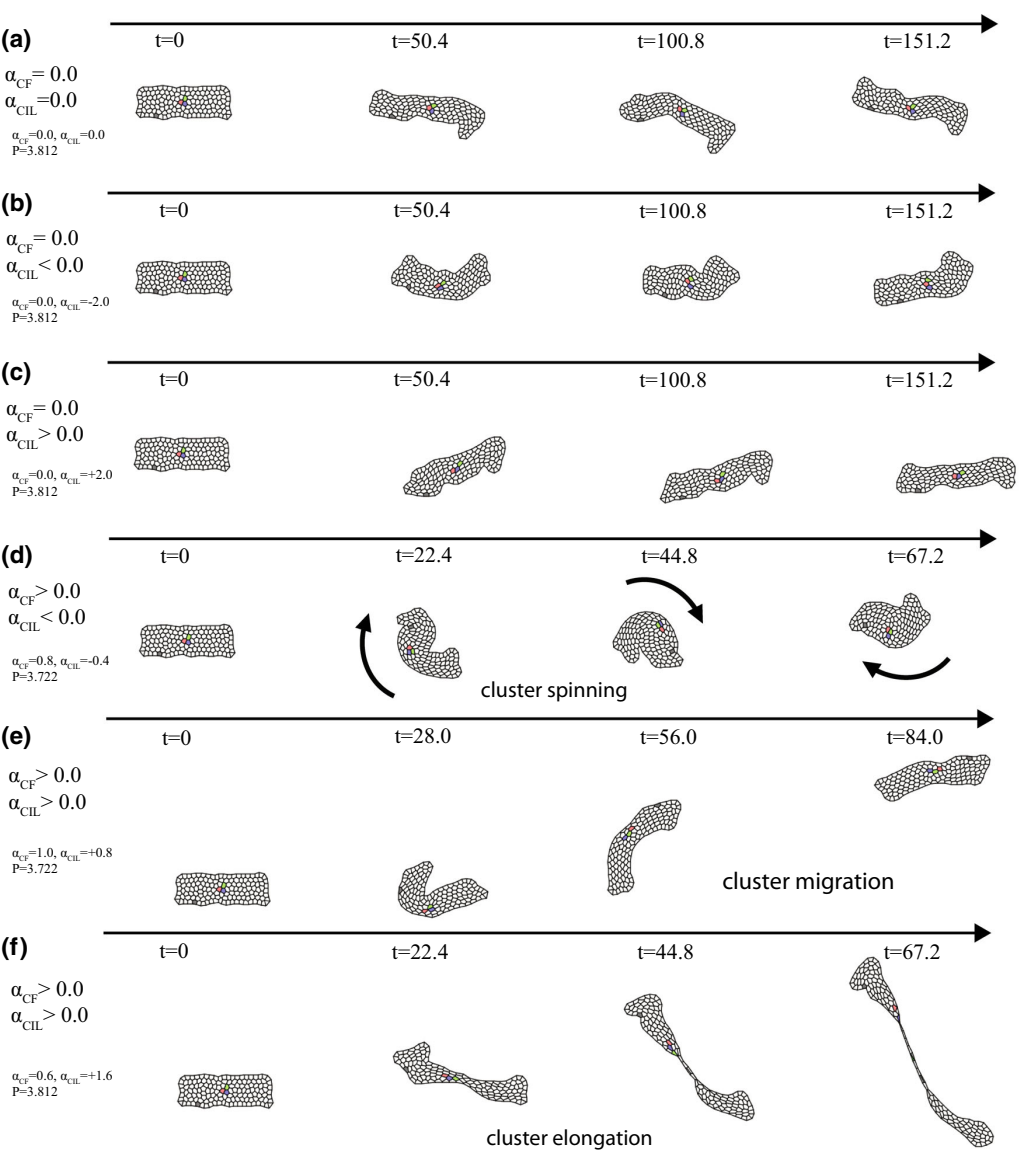
Simulation results for migrating epithelial cells in a free space. **a**–**c** For $\alpha _\mathrm{CF}=0.0$, characteristic dynamics is not observed. **d**–**f** For $\alpha _\mathrm{CF}>0.0$, epithelial cell clusters show various characteristic dynamics including **d** spinning, and **e** migration and **f** elongation. The parameter value set used for each figure is **a** $\alpha _\mathrm{CF}=0.0$, $\alpha _\mathrm{CIL}=0.0$ and $P=3.812$, **b** $\alpha _\mathrm{CF}=0.0$, $\alpha _\mathrm{CIL}=-2.0$ and $P=3.812$, **c** $\alpha _\mathrm{CF}=0.0$, $\alpha _\mathrm{CIL}=+2.0$ and $P=3.812$, **d** $\alpha _\mathrm{CF}=0.8$, $\alpha _\mathrm{CIL}=-0.4$ and $P=3.722$, **e** $\alpha _\mathrm{CF}=1.0$, $\alpha _\mathrm{CIL}=0.8$ and $P=3.722$, and **f** $\alpha _\mathrm{CF}=0.6$, $\alpha _\mathrm{CIL}=1.6$ and $P=3.812$. $K=1.0$

**Fig. 9:**
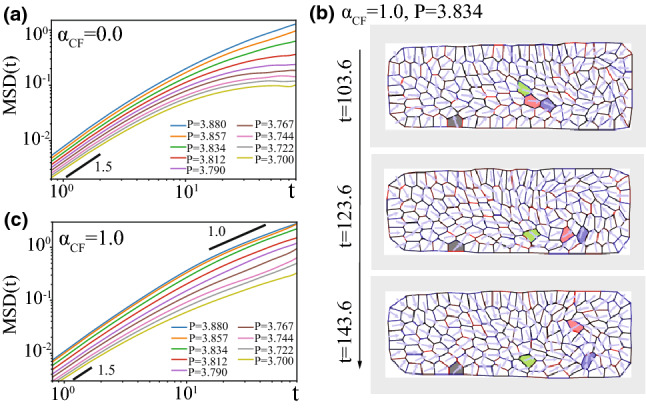
Simulation results corresponding to Fig. 4 in the presence of junctional tension fluctuation for $\alpha _\mathrm{CIL}=0.0$. **a** Mean square displacement for various shape index *P* for $\alpha _\mathrm{CF}=0.0$ and $\alpha _\mathrm{CIL}=0.0$. **b** Snapshots for $P=3.834$, $\alpha _\mathrm{CF}=1.0$ and $\alpha _\mathrm{CIL}=0.0$. **c** Mean square displacement for various shape index *P* for $\alpha _\mathrm{CF}=1.0$ and $\alpha _\mathrm{CIL}=0.0$. $K=4.0$

**Fig. 10:**
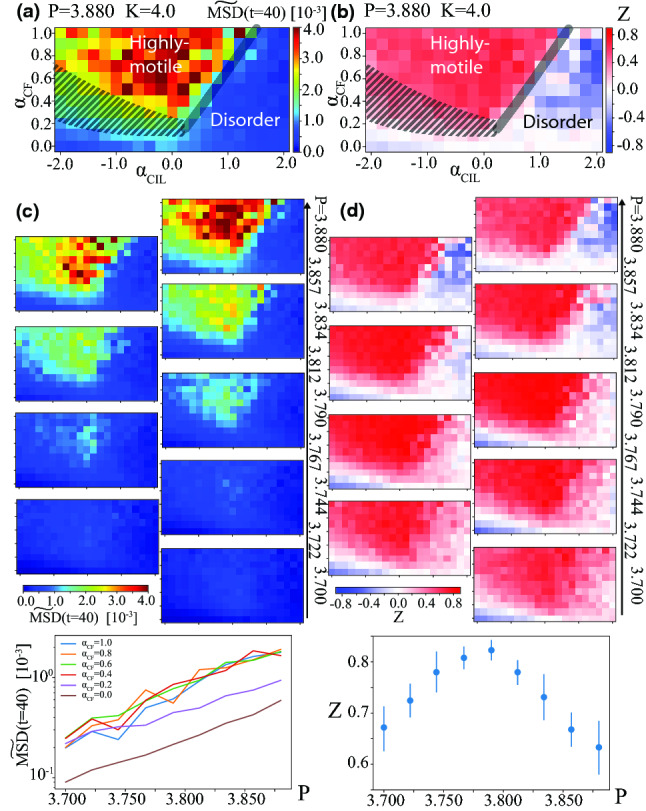
Simulation results corresponding to Fig. 5 in the presence of junctional tension fluctuation. **a** $\widetilde{\mathrm{MSD}}(t=40)$ against $\alpha _\mathrm{CIL}$ and $\alpha _\mathrm{CF}$ for the shape index $P=3.880$. **b** *Z* value, defined in Eq. 22 against $\alpha _\mathrm{CIL}$ and $\alpha _\mathrm{CF}$ for $P=3.880$. **c** $\widetilde{\mathrm{MSD}}(t=40)$ against $\alpha _\mathrm{CIL}$ and $\alpha _\mathrm{CF}$ for various *P*. The bottom graph plots $\widetilde{\mathrm{MSD}}(t=40)$ as a function of *P* for $\alpha _\mathrm{CIL}=0.0$ and various $\alpha _\mathrm{CF}$. **d** *Z* value, defined in Eq. 22 against $\alpha _\mathrm{CIL}$ and $\alpha _\mathrm{CF}$ for various *P*. The bottom graph plots *Z* value as a function of *P*; averaged among results for various $(\alpha _\mathrm{CIL}, \alpha _\mathrm{CF})$ for better statistics (within the range of $-0.4<\alpha _\mathrm{CIL}<0.4$ and $0.8< \alpha _\mathrm{CF}<1.0$.) K=4.0. Error bars indicate standard deviations

In this subsection, we briefly study epithelial cell dynamics without the system boundary and demonstrate the cluster dynamics. When $\alpha _\mathrm{CF}=0.0$, characteristic dynamics of a cluster is not observed for any $\alpha _\mathrm{CIL}$; the clusters just waver around (Fig. 7a–c). When $\alpha _\mathrm{CF}$ is enough large, as $\alpha _\mathrm{CF} > 0.2$, the cluster exhibits characteristic dynamics. For negative $\alpha _\mathrm{CIL}$, the cluster tends to spin (Fig. 7d). For positive $\alpha _\mathrm{CIL}$, the cluster tends to migrate or elongate, *i.e.* the two sides of a cluster travel to the different directions (Fig. 7e or f, respectively). These tendencies are roughly consistent with what we investigated for the case of freely migrating cells in Sect. 3.1. The coherent dynamics with apparent swirling for the freely migrating cells may correspond to the spinning in this case, whereas the stuck, or global polarly ordered flocking under a periodic boundary condition, may correspond to the cluster migration and elongation. It is apparently stochastic whether cluster migration or elongation takes place.

## Summary and discussion

In this article, we extended our previous theoretical investigation on dynamic self-organization of migrating cells through cell–cell contact communication [14] to cases with spatial confinement and/or epithelial integrity, and studied what coherent dynamics are caused and how this contributes to tissue dynamics. Epithelial monolayer dynamics were numerically simulated based on a mathematical model that combined self-propelled particle models with cell–cell contact communication for basal-side dynamics and the 2D cellular vertex model for apical-side dynamics.

Our main focus was whether and how the coherent dynamics due to cell–cell contact communication can help the cells overcome the suppression of motility by the constraints mentioned above. Both with and without epithelial integrity, we identified parameter regimes in which cells exhibited highly motile coherent dynamics, including swirling in the presence of contact following. We found that such coherent dynamics can help the cells overcome constraints due to spatial confinement and epithelial integrity and enhance the cell motility. This happened even when the parameter values of the vertex model (shape index) at the apical side were given to set the tissue in the solid regime.

By taking advantage of our model, we also investigated how the obtained coherent dynamics depend on the strength of the elastic connection between apical and basal sides *K* and the junctional tension fluctuation. Junctional tension fluctuation did not change the results significantly. Cell motility in the highly-motile regime, as measured by mean square displacement, depends on the strength *K* of the apico-basal connection. Furthermore, we demonstrated the potential behaviors of epithelial cell clusters without spatial confinement according to dynamic self-organization. Our simulation suggested that cell clusters adopt either spinning, migration, and elongation behavior.

Coherent rotational motions of migrating epithelial cells were observed in Ref. [20] by numerical simulations, under the assumption that each cell has a memory of past motile directions (with a finite memory time) and drives itself along the average of such motile directions in memory. The swirling behavior due to contact following which we observed here is distinct from such coherent rotation. Coherent rotation in Ref. [20] is a global rotation by which the migration direction of the cells follows the direction of the system boundary wall, whereas our swirling is a local circulation. It may be interesting for a future study to see what cell behaviors arise by a combination of these two different mechanisms of coherent dynamics. We expect that such study clarifies the relation between the apparent swirling dynamics observed in this article, which essentially requires contact following, and the local swirling dynamics due to CIL combined with the alignment of the polarity- to motility-directions [24].

To make the theoretical results comparable with various experiments using an epithelial monolayer, future studies will need the following technical improvements. In this article, we used much larger energy scale for the constraints of apical side area and perimeter than the basal driving force for migration such that, even in the presence of cell–cell communication, the cell area was not changed largely. However, in reality, each cell area in a monolayer can fluctuate largely and this may be important for phenomena such as collective oscillation [16]. In addition, our theoretical system comprises 140 cells, but this may be too small to compare with experimental systems required to study collective behavior. These settings were needed in this study because the illegal process of cellular vertex dynamics mentioned in the preface of Sect. 3 occurs more frequently for smaller apical energy scale and larger system size. By improving the technical details of the model and making the numerical simulation more stable, we will be able to reliably investigate wider varieties of epithelial cells dynamics.

In this article, we considered only the contact cell–cell communication through the basal side. However, in real tissues, other channels of cell–cell communication may be also important. Since cells are connected by the apical junctions, they can communicate through both chemical and mechanical signaling through apical side. Moreover, the cells can secret the chemicals to the extracellular environment which might facilitate communication between cells in a distance. Although the type of cell–cell communication considered in this article is limited, our results imply that dynamic self-organization due to cell–cell communication can overcome the suppression of motility by various constraints in a tissue, which could be extended to more generic cases.

## Figures and Tables

**Fig. 8:**
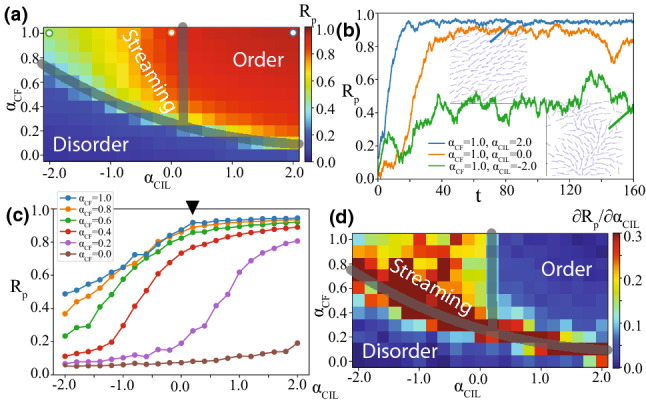
Simulation results for freely migrating cells in a periodic boundary. **a** Polar order parameter $\bar{R_p}$, defined in Eq. 23, against $\alpha _\mathrm{CIL}$ and $\alpha _\mathrm{CF}$. **b** Time evolution of instantaneous polar order $R_p(t)$ defined in Eq. 24 for $(\alpha _\mathrm{CIL},\alpha _\mathrm{CF})=(2.0, 1.0), (0.0, 1.0)$ and $(-2.0, 1.0)$. Insets: Corresponding snapshots. **c** Polar order parameter $\bar{R_p}$ as a function of $\alpha _\mathrm{CIL}$ for various $\alpha _\mathrm{CF}$. **d** $\partial \bar{R_p}/ \partial \alpha _\mathrm{CIL}$ against $\alpha _\mathrm{CIL}$ and $\alpha _\mathrm{CF}$

**Fig. 11:**
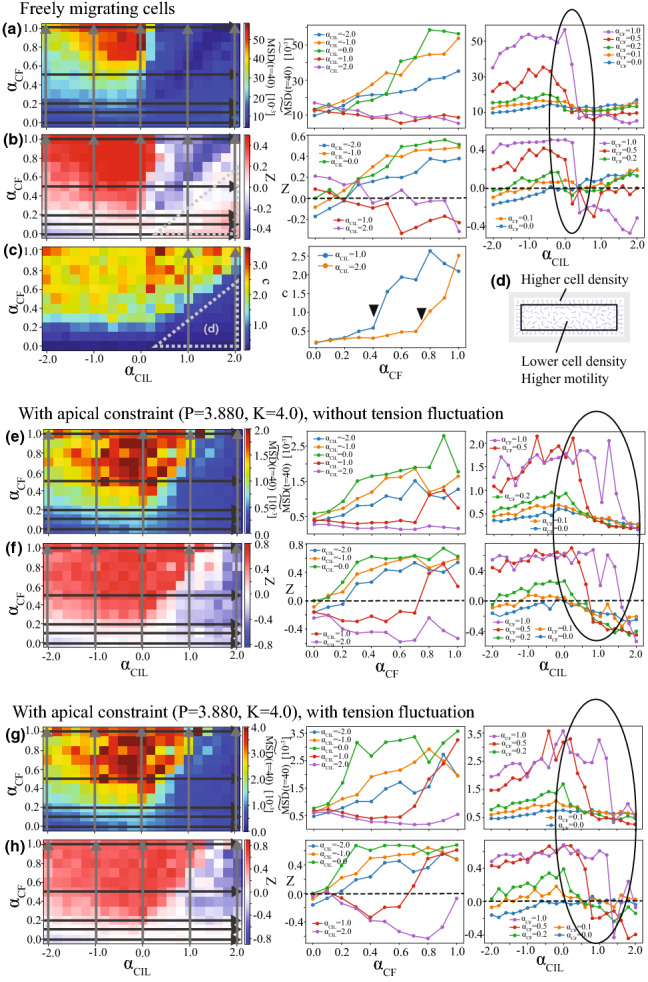
Summary of quantifications to determine state boundaries for the cases of freely migrating cells (**a**)–(**d**) and under apical constraint with (**e**, **f**) and without line tension fluctuation (**g**, **h**). **a**, **e**, **g** Mean square displacements at $t=40$. **b**, **f**, **h** *Z* values. **c** Degree of asymmetric cell distribution *c*. In each, left, center and right rows show the colormap over $(\alpha _\mathrm{CIL}, \alpha _\mathrm{CF})$, dependency on $\alpha _\mathrm{CF}$ for various given $\alpha _\mathrm{CIL}$ and $\alpha _\mathrm{CIL}$ for various given $\alpha _\mathrm{CF}$, respectively. The circles and inverse triangles highlight the state boundaries. **d** Sample snapshot of the regime where the outermost cells form shell-like layer

## A Freely migrating cells in a periodic boundary

In the previous work [14], we studied cellular dynamic self-organization in a periodic boundary and found that apparent global orientation of cell polarities is a useful measure to classify several states. In this appendix, we study the global orientational order of the similar system but with the small system size and parameter-value set used in the main text of this article ($N=140$ cells; *cf.*, in Ref. [14], we used $N=10,000-80,000$).

The results of the numerical simulations are given in Fig. 8. Figure 8a shows the polar order parameter

23 $$\begin{aligned} \bar{R_p} \equiv \left\langle \frac{1}{T_\mathrm{end}-T_\mathrm{ini}} \int ^{T_\mathrm{end}}_{T_\mathrm{ini}} dt \left| \frac{1}{N} \sum _{j=1}^{N}{\varvec{q}}_j (t) \right| ^2 \right\rangle \end{aligned}$$

against $\alpha _\mathrm{CIL}$ and $\alpha _\mathrm{CF}$ with $T_\mathrm{ini}=80$ and $T_\mathrm{end}=160$. $\langle \cdot \rangle$ means the ensemble average over 16 independent realizations. We find that as $\alpha _\mathrm{CF}$ is increased, the polar order arises.

Time evolution of instantaneous order parameter

24 $$\begin{aligned} R_p (t) \equiv \left| \frac{1}{N} \sum _{j=1}^{N} {\varvec{q}}_j (t) \right| ^2 \end{aligned}$$

of three representative cases (corresponding white points in Fig. 8a) are shown in Fig. 8b. For $\alpha _\mathrm{CIL}>0.2$, the stable polar order is established whereas, for $\alpha _\mathrm{CIL}<0.2$, the instantaneous order parameters fluctuates. Corresponding snapshots for $\alpha _\mathrm{CIL}=-2.0$ and $\alpha _\mathrm{CIL}=2.0$ are displayed as the insets, which again shows the clear difference; the case for $\alpha _\mathrm{CIL}<0.2$ lacks the clear order which is seen for $\alpha _\mathrm{CIL}>0.2$ but exhibits converging streams. Therefore, it is expected that there is the boundary between the genuinely ordered regime and dynamic streaming regime around $\alpha _\mathrm{CIL} \sim 0.2$. In Ref. [14], by carefully studying the systems size dependency, we realized that the dependence of apparent global order parameter $\bar{R_p}$ on the parameter such as $\alpha _\mathrm{CIL}$ is a good measure to identify such state boundary. Hence, we plot $\bar{R_p}$ against $\alpha _\mathrm{CIL}$ in Fig. 8c and find that $\alpha _\mathrm{CIL}$-dependence of $\bar{R_p}$ changes around $\alpha _\mathrm{CIL} \sim 0.2$. To see this fact explicitly, we plot $\partial \bar{R_p}/ \partial \alpha _\mathrm{CIL}$ in Fig. 8d. Indeed, for $\alpha _\mathrm{CF}>0.2$, $\partial \bar{R_p}/ \partial \alpha _\mathrm{CIL}$ starts to rapidly increase near $\alpha _\mathrm{CIL} \sim 0.2$ as $\alpha _\mathrm{CIL}$ is decreased. Collectively, we determine the state boundaries among disorder, order and streaming states as shown by the solid curves in Fig. 8a, d.

## B Results for the case with line-tension fluctuation at the apical cell–cell junctions

Figures 9 and 10 plot the results of the numerical simulations with the junctional tension fluctuation in the vertex model at the apical side, in the similar manner with Figs. 4 and  5, respectively. Qualitatively, all the features seen in the case without tension fluctuation are maintained. The difference is observed only in the quantitative features. In particular, the shape index *P* at the solid-fluid phase transition seems to be shifted to the lower *P*, around $P\sim 3.790$, as shown in Fig. 9a. (*cf.* In the absence of the tension fluctuation, transition occurs at $P=3.812$.) The optimum *P* giving the largest *Z* value is also shifted similarly, as shown in Fig. 10d bottom.

## C How the state boundaries are determined

This appendix, together with Fig. 11, summarizes how we have divided the states in the main text of this article both for freely migrating cells (Fig. 11a–d) and the cells under apical constraints (Fig. 11e–h). In this article, we focused on the motility of the cells quantified by mean square displacements (MSD). As shown in Fig. 11a, e, g and highlighted by the circles, we find that, when we increase the $\alpha _\mathrm{CIL}$ from $-2.0$ to $+2.0$, the mean square displacement at the fixed time, $\mathrm{MSD}(t=40.0)$, suddenly drops at a certain $\alpha _\mathrm{CIL}$ for $\alpha _\mathrm{CF} \ge 0.3$; for example, at $\alpha _\mathrm{CIL} \sim 0.2$ for $\alpha _\mathrm{CF}=1.0$ (purple curve) in Fig. 11a right. These sudden drops of the motility corresponds to the shift from the state with high motility (Fig. 2d, e for freely migrating cell case) to that with low motility (Fig. 2a–c for freely migrating cell case). In this article, the former state is called the highly-motile state whereas the later state(s) called the disorder or stuck state. To further explicitly quantify these states, we used the quantity *Z* in Eq. (22), which is similar to measuring $\mathrm{MSD}$ itself at a fixed time but takes care of the following issues:

- Depending on the constraint situations (freely migrating or under apical constraints) and parameter values, the $\mathrm{MSD}$ value at the fixed time in the absence of any intercellular communication, i.e., $(\alpha _\mathrm{CF},\alpha _\mathrm{CIL})=(0, 0)$, varies. To avoid this, $\mathrm{MSD}$ value in the absence of any intercellular communication was subtracted.
- The absolute value of $\mathrm{MSD}$ varies by orders of magnitudes depending on the situations. To avoid this, in the definition of *Z*, the value was rescaled such that it ranges within $-1.0$ and $+1.0$.
- As shown in Fig. 3c, most of the cells are apparently less motile. In the definition of *Z*, we only used the cells with the top 10 percent of the $\mathrm{MSD}$ over all the samples.

Empirically, this definition of *Z* works well to classify the high- and low-motility states; As shown in Fig. 11b, f, h, around the state boundary, the sign of this *Z*’s value flips. According to this empirical characteristics, we decided to use the sign of *Z* to define the boundary between the high- and low-motility regimes.

Be cautious that we have another state boundary for the cases with freely migrating cells as follows. Plotting the degree of asymmetric cell distribution *c* exhibits the sudden changes of the slopes over $\alpha _\mathrm{CF}$, indicated by the inverse triangles in Fig. 11c right. In the parameter region where $\alpha _\mathrm{CF}$ is lager than this boundary, we find the low *MSD* or negative *Z* as well as high value of *c*. This means that, in this regime, the cells are not only asymmetrically distributed but also less motile. Such qualitative difference around this boundary is consistent with the difference between the states shown in Fig. 2b, where no asymmetric distribution is observed, and Fig. 2c, where the cells are asymmetrically distributed and stuck at a system edge. In the state shown in Fig. 2b, apparently the outermost cells are forming high-density shell-like layer. When the cells form such high-density shell-like layer, the motility becomes higher due to the lower density in the bulk regions as illustrated in Fig. 11d. This is why the positive *Z* takes place at the bottom right region of Fig. 11b left. We excluded this positive-*Z* region from the above arguments to dissect the highly motile and the other states.

There are other ways to define the highly-motile states. In Ref. [24], so-called cage relative mean square displacement and the vortex density are used to define it. This definition can further dissect the global and local coherent motions. Although this article did not discuss this aspect, it is a possible future direction to investigate how the pair of contact following and contact inhibition/attraction of locomotion, which are the types of intercellular communication used in this article, can affect the locality of coherent motility of cells.

## References

[1] Wedlich-Söldner RR, Betz T (2018). Self-organization: the fundament of cell biology. Philos. Trans. R. Soc. B.

[2] Santos AX, Liberalli P (2019). From single cells to tissue self-organization. FEBS J..

[3] Collinet C, Lecuit T (2021). Programmed and self-organized flow of information during morphogenesis. Nat. Rev. Mol. Cell Biol..

[4] Mayor R, Carmona-Fontaine C (2010). Keeping in touch with contact inhibition of locomotion. Trends Cell Biol..

[5] Carmona-Fontaine C, Matthews HK, Kuriyama S, Moreno M, Dunn GA, Parsons M, Stern CD, Mayor R (2008). Contact inhibition of locomotion in vivo controls neural crest directional migration. Nature.

[6] Theveneau E, Marchant L, Kuriyama S, Gull M, Moepps B, Parsons M, Mayor R (2010). Collective chemotaxis requires contact-dependent cell polarity. Dev. Cell.

[7] A. Szabó, E. Theveneau, M. Turan, R. Mayor Neural crest streaming as an emergent property of tissue interactions during morphogenesis. PLoS Comput. Biol. **15**, e1007002 (2019) 10.1371/journal.pcbi.1007002 PMC6497294 31009457

[8] Li Y, Vieceli FM, Gonzalez WG, Li A, Tang W, Lois C, Bronner ME (2019). In vivo quantitative imaging provides insights into trunk neural crest migration. Cell Rep..

[9] Gregor T, Fujimori K, Masaki N, Sawai S (2010). The onset of collective behavior in social amoebae. Science.

[10] Kuwayama H, Ishida S (2013). Biological soliton in multicellular movement. Sci. Rep..

[11] T. Fujimori, A. Nakajima, N. Shimada, S. Sawai, Tissue self-organization based on collective cell migration by contact activation of locomotion and chemotaxis. PNAS **116**, 4291–4296 (2019) 10.1073/pnas.1815063116 PMC6410881 30782791

[12] Hayakawa M, Hiraiwa T, Wada Y, Kuwayama H, Shibata T (2020). Polar pattern formation induced by contact following locomotion in a multicellular system. eLife.

[13] Hiraiwa T (2019). Two types of exclusion interactions for self-propelled objects and collective motion induced by their combination. Phys. Rev. E.

[14] Hiraiwa T (2020). Dynamic self-organization of idealized migrating cells by contact communication. Phys. Rev. Lett..

[15] Saw TB, Doostmohammadi A, Nier V, Kocgozlu L, Thampi S, Toyama Y, Marcq P, Lim CT, Yeomans JM, Ladoux B (2017). Topological defects in epithelia govern cell death and extrusion. Nature.

[16] G. Peyret, R. Mueller, J. d’Alessandro, S. Begnaud, P. Marcq, R.-M. Mege J. M. Yeomans, A. Doostmohammadi, B. Ladoux, Biophys. J. **117**, 464 (2019) 10.1016/j.bpj.2019.06.013 PMC6697349 31307676

[17] Gilbert SF (2010). Developmental Biology.

[18] Nagai T, Honda H (2001). Philos. Mag. Part B.

[19] Hiraiwa T, Nagamatsu A, Akuzawa N, Nishikawa M, Shibata T (2014). Relevance of intracellular polarity to accuracy of eukaryotic chemotaxis. Phys. Biol..

[20] Li B, Sun SX (2014). Coherent motions in confluent cell monolayer sheets. Biophys. J..

[21] Bi D, Yang X, Marchetti MC, Manning ML (2016). Motility-driven glass and jamming transitions in biological tissues. Phys. Rev. X.

[22] Giavazzi F, Paoluzzi M, Macchi M, Bi D, Scita G, Manning ML, Cerbino R, Marchetti MC (2018). Flocking transitions in confluent tissues. Soft Matter.

[23] Barton DL (2017). Active Vertex Model for cell-resolution description of epithelial tissue mechanics. PLoS Comput. Biol..

[24] Lin S-Z, Ye S, Xu G-K, Li B, Feng X-Q (2018). Dynamic migration modes of collective cells. Biophys. J ..

[25] Czajkowski M, Sussman DM, Marchetti MC, Manning ML (2019). Glassy dynamics in models of confluent tissue with mitosis and apoptosis. Soft Matter.

[26] Sun Z, Amourda C, Shagirov M, Hara Y, Saunders TE, Toyama Y (2017). Basolateral protrusion and apical contraction cooperatively drive Drosophila germ-band extension. Nat. Cell Biol..

[27] C. Carmona-Fontaine, E. Theveneau, A. Tzekou, M. Tada, M. Woods, K.M. Page, M. Parsons, J.D. Lambrisand, R. Mayor, Complement fragment C3a controls mutual cell attraction during collective cell migration. Dev. Cell **21**, 1 (2011) 10.1016/j.devcel.2011.10.012 PMC3272547 22118769

[28] R.A. Desai, S.B. Gopal, S. Chen, C.S. Chen, Contact inhibition of locomotion probabilities drive solitary versus collective cell migration. J. R. Soc. Interface **10**, 20130717 (2013) 10.1098/rsif.2013.0717 PMC3785843 24047876

[29] B. Lin, T. Yin, Y.I. Wu, T. Inoue, A. Levchenko, Interplay between chemotaxis and contact inhibition of locomotion determines exploratory cell migration. Nat. Commun. **6**, 6619 (2015) 10.1038/ncomms7619 PMC4391292 25851023

[30] Li D, Wang Y (2018). Coordination of cell migration mediated by site dependent cell-cell contact. PNAS.

[31] Tanida S, Furuta K, Nishikawa K, Hiraiwa T, Kojima H, Oiwa K, Sano M (2020). Gliding filament system giving both orientational order and clusters in collective motion. Phys. Rev. E.

[32] T. Hiraiwa, R. Akiyama, D. Inoue, A. Md. R. Kabir, A. Kakugo, Mono-polar clustering of self-propelled particles through left-right asymmetry. arXiv:2101.02130

[33] Romanczuk P, Couzin ID, Schimansky-Geier L (2009). Collective motion due to individual escape and pursuit response. Phys. Rev. Lett..

[34] Couzin ID, Krause J, James R, Ruxton GD, Franks NR (2002). Collective memory and spatial sorting in animal groups. J. Theor. Biol..

[35] Barberis L, Peruani F (2016). Large-scale patterns in a minimal cognitive flocking model: incidental leaders, Nematic patterns, and aggregates. Phys. Rev. Lett..

[36] Peruani F (2017). Hydrodynamic equations for flocking models without velocity alignment. J. Phys. Soc. Jpn..

[37] Grossman D, Aranson IS, Jacob EB (2008). Emergence of agent swarm migration and vortex formation through inelastic collisions. New J. Phys..

[38] Hanke T, Weber CA, Frey E (2013). Understanding collective dynamics of soft active colloids by binary scattering. Phys. Rev. E.

[39] Bertin E, Droz M, Grégoire G (2006). Boltzmann and hydrodynamic description for self-propelled particles. Phys. Rev. E.

[40] R. Grossmann, L.S.-Geier, P. Romanczuk, Self-propelled particles with selective attraction-repulsion interaction: from microscopic dynamics to coarse-grained theories. New J. Phys. **15**, 085014 (2013)

[41] Camley BA, Zhang Y, Zhao Y, Li B, Ben-Jacob E, Levine H, Rappel W-J (2016). Polarity mechanisms such as contact inhibition of locomotion regulate persistent rotational motion of mammalian cells on micropatterns. PNAS.

[42] Farhadifar R, Röper J-C, Aigouy B, Eaton S, Jülicher F (2007). Curr. Biol..

[43] Staple DB, Farhadifar R, Röper J-C, Aigouy B, Eaton S, Jülicher F (2010). Euro. Phys. J. E.

[44] Sato K, Hiraiwa T, Maekawa E, Isomura A, Shibata T, Kuranaga E (2015). Nat. Commun..

[45] Sato K, Hiraiwa T, Shibata T (2015). Phys. Rev. Lett..

[46] T. Hiraiwa, E. Kuranaga, T. Shibata, Front. Cell Dev. Biol. **5**, 66 (2017) 10.3389/fcell.2017.00066 PMC5516087 28770197

[47] Hiraiwa T, Wen F, Kuranaga E, Shibata T (2019). Symmetry.

[48] T. Yamamoto, T. Hiraiwa, T. Shibata, Phys. Rev. Res. **2**, 043326 (2020)

[49] Weliky M, Oster G (1990). The mechanical basis of cell rearrangement. I. Epithelial morphogenesis during Fundulus epiboly. Development.

[50] Fily Y, Marchetti MC (2012). Athermal phase separation of self-propelled particles with no alignment. Phys. Rev. Lett..

[51] G.S. Redner, M.F. Hagan, A. Baskaran, Structure and dynamics of a phase-separating active colloidal fluid. Phys. Rev. Lett. **110**, 055701 (2013) 10.1103/PhysRevLett.110.055701 23414035

[52] Bi D, Lopez JH, Schwarz JM, Manning ML (2015). A density-independent rigidity transition in biological tissues. Nat. Phys..

